# Bladder cancer: worse survival in women from deprived areas

**DOI:** 10.1038/sj.bjc.6601847

**Published:** 2004-04-27

**Authors:** A Moran, A-M Sowerbutts, S Collins, N Clarke, R Cowan

**Affiliations:** 1 Centre for Cancer Epidemiology, University of Manchester, Christie Hospital NHS Trust, Manchester M20 4QL, UK; 2 Christie Hospital NHS Trust, Manchester M20 4BX, UK

**Keywords:** bladder cancer, survival, stage, sex, deprivation

## Abstract

In a case-note review of 120 women and 227 men presenting with muscle-invasive bladder tumours in 1998, survival was worse for women in 3 years of follow-up, with the greatest difference, of 19.9%, at 6 months. For more deprived women, 6-month survival was 52.3%, and 32 (37.2%) presented with advanced disease, compared with 73.5%, and three (8.8%) for less deprived women.

Bladder cancer is the only common cancer for which women have worse prognosis than men as reported in the UK, continental Europe and North America (Micheli *et al*, 1998; Coleman *et al*, 1999; Ries *et al*, 1999). Studies based on routine registry data from the Netherlands and the US have reported later stage at diagnosis for women than for men and worse stage-specific survival (Fleshner *et al*, 1996; Mungan *et al*, 2000a, 2000b). These findings are unexplained. We report the results of a case-note review of patients with newly diagnosed muscle-invasive bladder cancers to explore the reasons for differences in survival by sex.

## MATERIALS AND METHODS

Patients were eligible if they were diagnosed with a muscle-invasive bladder carcinoma in 1998, were residents of Greater Manchester or Lancashire (GML) and had no previous history of bladder cancer.

All patients in GML diagnosed with a new bladder tumour in 1998 were identified from the database held at the North Western Cancer Registry, which receives information from all hospitals and pathology laboratories in GML, and death details from the Office of National Statistics (ONS). Pathology reports received by the Registry were reviewed for evidence of muscle-invasive disease (MID). Case notes were requested if pathology confirmed or suggested MID, or information was incomplete. In order to identify patients with a diagnosis of MID based on clinical or radiological, rather than histological, findings, the case notes of patients not in the above categories but who either underwent radiotherapy in 1998 or who died between 1st January 1998 and 31st December 2001 were also requested.

Patients were included in the study if they had MID confirmed on histology, or they fulfilled one of the following conditions: residual mass present following resection of a bladder tumour; bladder mass on scan or ultrasound with bladder cancer considered the most likely diagnosis; or a large necrotic bladder tumour on cystoscopy which was not investigated further.

The dates of death were obtained from the Registry, and deaths were classified into those due to bladder cancer or other causes, using death certificates and case notes. Stage was determined using pathology reports and case notes, with clinical staging used for all analyses. Initially, tumours were staged as T2, T3, T4a or T4b, but for analyses were classified into advanced (T4b and/or M1) or nonadvanced. Details of histology and grade were obtained from pathology reports.

Material deprivation was measured using the Townsend score of the enumeration district (ED) in which the patient lived, determined from Registry postcode details. Scores were classified into five groups based on quintiles for England.

Frequency distributions were compared using *χ*^2^ tests and *t*-tests. Cause-specific survival rates for bladder cancer were estimated using the Kaplan–Meier method, with survival measured from the date of first tumour resection until the date of death, and censoring on the earlier of date of death due to other causes, or 30 June 2002; comparisons were made using the log-rank test. All significance tests were two-sided.

## RESULTS

A total of 1190 patients diagnosed with a first bladder tumour in 1998 were identified on the Registry database. Case notes of 754 patients with suspected MID were requested and 676 (90%) reviewed; 347 were confirmed as having MID without a history of bladder cancer. In all, 329 patients were excluded: 34 in fact had a bladder tumour prior to 1998, 14 had tumours other than carcinomas, 29 had insufficient information on which to base a diagnosis, nine had insufficient information to stage and 243 had superficial disease.

Of the study population of 347, 120 (34.6%) were female. The median age was 75.5 in women and 72.5 in men (*t*=1.271; 345 df; *P*=0.205). In all, 78.3% of women and 79.7% of men had either grade 3 or grade-unknown tumours. A total of 35 (29.2%) women presented with advanced disease compared with 33 (14.5%) men (*χ*^2^=10.66; 1 df; *P*<0.01). Compared with men, women were under-represented in the two least deprived quintiles (1+2) and quintile 4; this difference was not statistically significant (*χ*^2^=7.987; 4 df; *P*=0.092).

**Figure 1:**
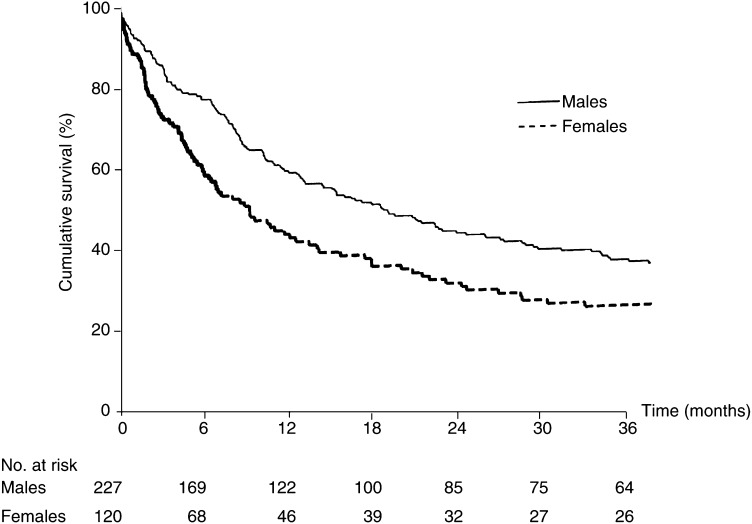
Survival from bladder cancer in males and females.

The difference in survival between men and women was 10.9% 3 months after diagnosis (Figure 1

), was greatest after 6 months (19.9%), thereafter decreasing to 15.8% at 12 months and 9.9% at 3 years. In total, 49 women and 48 men died from bladder cancer in the 6-month period following diagnosis (log-rank *χ*^2^=14.74; 1 df; *P*<0.01).

When women were categorised according to whether they were from less deprived (quintiles 1+2) or more deprived areas (the remaining quintiles), the 6-month survival was 73.5% for the less deprived compared with 52.3% for the more deprived (*χ*^2^=4.83; 1 df; *P*<0.05). Three (8.8%) women from less deprived and 32 (37.2%) from more deprived areas presented with advanced disease (*χ*^2^=9.50; 1 df; *P*<0.01) (Table 2). There was no difference in the age distribution of women from the two deprivation categories. Neither survival nor stage varied by deprivation for men (Tables 1

**Table 1:** 6-month survival rates (%) for each deprivation quintile

**Quintile**	**Male**	**Female**
1	79.5	73.7
2	85.2	73.3
3	78.1	60.9
4	72.8	45.8
5	78.5	50.0
Total	78.4	58.4

and 2

**Table 2:** Number (%) with advanced disease for each deprivation quintile

**Quintile**	**Male**	**Female**
1	8 (17.8%)	3 (15.8%)
2	5 (11.9%)	0 (0.0%)
3	4 (12.1%)	9 (31.0%)
4	7 (11.9%)	9 (36.0%)
5	9 (18.8%)	14 (43.8%)
Total	33 (14.5%)	35 (29.2%)

).

## DISCUSSION

Women had worse survival than men and were twice as likely to present with advanced disease. Women from more deprived areas had worse survival and were more likely to present with advanced tumours than men or women from less deprived areas.

This population-based study reflects the experience of all patients diagnosed with new muscle-invasive bladder cancer in GML in 1998. Clinical staging was used, as less than 20% of subjects underwent cystectomy: compared with staging based on examination of cystectomy specimens, this results in the understaging of a considerable proportion of tumours (Paik *et al*, 2000). However, such misclassification is an implausible explanation for our finding that advanced disease was proportionally twice as common in women as men, as one would have to postulate that clinical and radiological investigations were systematically better at identifying the early signs of advanced disease in women than in men.

We found that the difference in survival by sex was greatest 6 months after diagnosis. Other studies have reported the difference as greatest at 1 year after diagnosis, but none provide results for periods of less than 1 year (Micheli *et al*, 1998; Coleman *et al*, 1999; Ries *et al*, 1999; Mungan *et al*, 2000b). Our results underline the importance of undertaking a more detailed analysis. The pattern of survival differences over time in this study – a substantial difference within 3 months of diagnosis, reaching a maximum within 6 months – suggests that the most likely explanation for worse survival in women is that they more commonly present with advanced disease.

When the superficial tumours (*n*=243) are added to our study population, the difference between the sexes in 1-year survival is 9%, similar to that for England and Wales (Coleman *et al*, 1999). Studies from the US and the Netherlands have reported later stage distribution in women than men and 1-year stage-specific survival rates 7–16% higher for men (Ries *et al*, 1999; Mungan *et al*, 2000a, 2000b). Small decreases in bladder cancer survival (5% in men and 7% in women) with increasing deprivation have been reported for England and Wales (Coleman *et al*, 1999). The only published results on the relationship between stage and deprivation are from a US hospital-based study, which reported that patients from low-income neighbourhoods were twice as likely to present with stage IV bladder cancers as those from high-income neighbourhoods; results by sex were not presented (Fleshner *et al*, 1996).

In this study, the finding of worse survival in females appears to be confined to women from more deprived areas. The 6-month survival for this group was 26% lower than for men, compared with a difference of less than 5% between men and women from less deprived areas. Three out of eight women with MID from more deprived areas presented with advanced disease: a much higher proportion than for men, or less deprived women. Later stage at diagnosis may be due to faster growing tumours or longer delay in diagnosis and treatment. Although it is not possible in our study to determine which of these is the main explanation for later stage disease in deprived women, longer delay seems the most plausible. Thus, women from poorer areas with haematuria may be more likely to delay attending their GP, or symptoms suggestive of urinary tract infection may be more common among this group, which could result in delay in referral to a urologist. Further research is needed to determine how these health inequalities can be eliminated.
